# Microwave-Assisted Superheating and/or Microwave-Specific Superboiling (Nucleation-Limited Boiling) of Liquids Occurs under Certain Conditions but is Mitigated by Stirring

**DOI:** 10.3390/molecules201219793

**Published:** 2015-12-04

**Authors:** Anthony Ferrari, Jacob Hunt, Albert Stiegman, Gregory B. Dudley

**Affiliations:** Department of Chemistry and Biochemistry, Florida State University, Tallahassee, FL 32306-4390, USA; af11j@my.fsu.edu (A.F.); jhunt@chem.fsu.edu (J.H.)

**Keywords:** superheating, microwave, reflux, temperature, boiling point

## Abstract

Temporary superheating and sustained nucleation-limited “superboiling” of unstirred liquids above the normal atmospheric boiling point have been documented during microwave heating. These phenomena are reliably observed under prescribed conditions, although the duration (of superheating) and magnitude (of superheating and superboiling) vary according to system parameters such as volume of the liquid and the size and shape of the vessel. Both phenomena are mitigated by rapid stirring with an appropriate stir bar and/or with the addition of boiling chips, which provide nucleation sites to support the phase-change from liquid to gas. With proper experimental design and especially proper stirring, the measured temperature of typical organic reaction mixtures heated at reflux will be close to the normal boiling point temperature of the solvent, whether heated using microwave radiation or conventional convective heat transfer. These observations are important to take into consideration when comparing reaction rates under conventional and microwave heating.

## 1. Introduction

Accurate temperature measurements are critical to understanding thermochemical processes. While this can be easily done for conventional convective heating using thermometers and thermocouples, it is not so easily done under microwave heating due to interactions of the radiation field with standard thermometers. There are two common approaches to measuring temperature inside the microwave cavity. One is to use remote, infrared (IR) sensors that measure the black-body emission of the system and derive the temperature from that. The second is the use of fiber optic thermometers that are unaffected by the radiation field and can provide accurate temperature determination inside the cavity. Ultimately, all temperature measurements are standardized against reproducible, physical phenomena, such as the melting and boiling points of water (*cf.* Centigrade scale). Even when accurate bulk temperature is determined, however, one must recognize and consider that microwave radiation creates heat through mechanisms that are distinct from those of convective heating, which potentially can result in selective heating and inhomogeneous temperature distributions that cannot be detected by bulk temperature measurements. Inhomogeneous temperature distributions in selectively heated systems must be inferred from other physical properties of the system. Reliable determinations of bulk temperature inside the microwave cavity are critical to understanding the unique impacts of microwave heating on thermochemical processes.

**Scheme 1:**
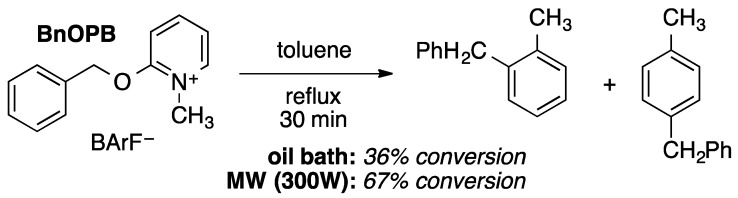
Recapitulation of thermal Friedel–Crafts benzylation reactions conducted in refluxing toluene (reprinted from [2]). Under otherwise-identical conditions, the reaction in which reflux was sustained by application of microwave radiation was faster. Both reaction mixtures were visibly homogeneous and vigorously stirred.

We reported [1]—and later quantified [2,3]—microwave-specific rate accelerations of organic reactions due to selective heating of polar solutes dissolved in nonpolar solvents. Many reports of such phenomena have been dismissed as artifacts of improper temperature measurements [4], and our series of papers stimulated considerable interest [5,6,7] and some controversy [8,9,10,11]. Much of the controversy related to confusion over the distinction between heat and temperature, and to the challenges of accurately measuring bulk temperature of microwave-heated solutions. To the latter point, we use internal fiber optic temperature probes, external infrared sensors, and/or thermal imaging cameras to record system temperatures in our experiments, and finally, we conducted experiments in which the physical boiling point of the solvent (toluene, at atmospheric pressure) determined the bulk solution temperature (Scheme 1).

The specific experiments recounted in Scheme 1 unambiguously point to a difference between microwave and convective heating on this reaction, but they do not alone clarify the origin of the difference. Based on this and a series of related experiments [1,2], we attributed the difference to selective heating of the ionic solute (BnOPB) in the microwave reactor, which perturbs thermal equilibrium between the solute and the bulk solvent (toluene). Interactions between microwave radiation and the ionic solute convert microwave electromagnetic energy into thermal energy (heat) within nanometer-sized solute domains, resulting in thermal reactivity of the solute that exceeds what would be expected based on the observable temperature of the bulk solvent. A more detailed explanation of the underlying theory can be found in our recent Perspective Article on microwave-specific reaction rate enhancement [12].

**Figure 1:**
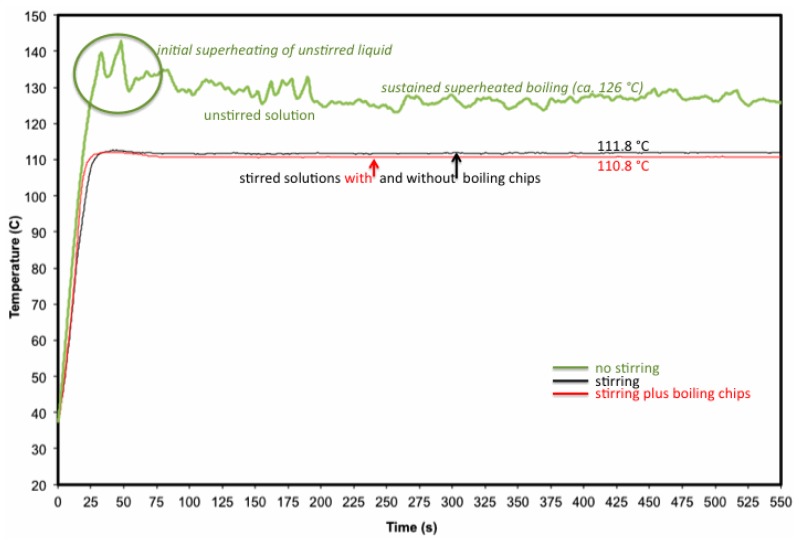
Plots of temperature over time for refluxing solutions of BnOPB in toluene under microwave heating (reprinted from [9]). The top (**green**) line shows the measured bulk temperature profile in the absence of stirring. Initial superheating (**circled**) is followed by sustained superboiling of the unstirred liquid. Superboiling was visibly chaotic, consistent with the erratic temperature profile. The lower two lines show the measured bulk temperature profiles of stirred solutions, either with or without boiling chips. These refluxing solutions appeared qualitatively similar to refluxing solutions under conventional heating to which they were compared.

An alternative potential explanation of the facts laid out in Scheme 1 is that microwave-specific solvent “superheating” [8] elevated the temperature of the toluene solution significantly above its atmospheric boiling point. Superheating phenomena in stirred liquids under microwave heating had been regarded as negligible [13], but then they were later reported and described by the same authors as being potentially confounding “even (when) applying vigorous stirring” [14]. In light of conflicting reports and confusion surrounding various potential forms of superheating in microwave experiments, we have examined the issue more closely. We immediately ruled out this alternative explanation of the observations recounted in Scheme 1 by directly measuring *in situ* the reflux temperature of our (stirred) reaction mixture (Figure 1, black line) [2,8]. Our investigations and observations will help bring clarity to the confusions surrounding the various forms of microwave superheating.

As noted above, superheating phenomena were negligible in our experiments involving stirred liquids (Figure 1, black line). What can also be seen in Figure 1 (green line), however, is that *in the absence of stirring*, we were able to document two related thermal events in rapid succession: superheating [15,16] and “superboiling” (nucleation-limited boiling [17]) of our toluene solution. These events—temporary superheating and sustained superboiling—have seemingly been conflated in some of the microwave chemistry literature, but we wish to make a clear distinction between the two. For example, only the latter is microwave-specific.

Superheating of liquids above the standard boiling point can be achieved without the use of microwave energy, although microwave heating facilitates the process. The temperature of a liquid can be elevated to above its boiling point, provided that care is taken to avoid nucleation sites that could help trigger the liquid→gas phase change. The superheated liquid will exist in this metastable state until it is perturbed, resulting in the formation of the initial gas bubble(s). Once boiling is initiated, however, the metastable liquid ceases to exist, and excess heat is quickly (and sometimes violently) liberated to the surroundings. In conventionally heated systems, the bulk temperature of the remaining liquid rapidly regresses to the normal boiling point expected for the ambient atmospheric conditions.

Superboiling (nucleation-limited boiling), in contrast to solvent superheating, has been reported as a microwave-specific phenomenon related to the inability of the bulk solvent to undergo a liquid→gas phase change fast enough to remove the radiation-generated bulk heat. This novel phenomenon has also been called “super-heated boiling” [18] or “superheating” [8,19], although the latter term is perhaps best reserved for the more general (and not microwave-specific) phenomenon described in the previous paragraph. We prefer the term “*superboiling*” for its simplicity and for how it reflects the distinction from conventional superheating of static liquids.

Like other reported microwave-specific phenomena, superboiling is perhaps neither recognized nor accepted broadly within in the organic community. As with much of the early literature on microwave chemistry and the effects of microwave heating, early reports of superboiling may be subject to skepticism owing to conflicting data and to the fundamental challenges associated with reproducibility and accurate temperature measurement. In our opinion, it can be difficult for the casual reader to discern which reported phenomena are likely to be reproducible and which are likely experimental artifacts. As noted above, we previously documented superheating and superboiling in unstirred toluene solutions, and we showed that vigorous stirring ameliorated both of these effects in toluene. Here we report the results of experiments using common alcoholic solvents. Taken together, our observations qualitatively validate previous reports of superboiling while underscoring the importance of potentially overlooked experimental design details on reaction outcomes.

## 2. Experimental Designs

All reflux experiments and measurements were conducted on a 30-mL volume of alcohol solvent in a 50-mL quartz round-bottom flask with a 24/40 joint connected to a reflux condenser, except where noted. Heating was accomplished using a CEM Discover (R) SP2 2.45 GHz microwave system operating at 75 W of applied power. Liquids were stirred, where noted, on the highest stirring setting possible in the CEM reactor system. Reagent-grade methanol, ethanol, and isopropyl alcohol were used as received.

The temperatures of the liquids were measured internally with a Neoptix (R) fiber optic temperature probe that was interfaced with the microwave, except where noted. The fiber optic thermometer was calibrated against a NIST traceable thermocouple accurate to ±0.001 °C.

## 3. Results and Discussion

**Figure 2:**
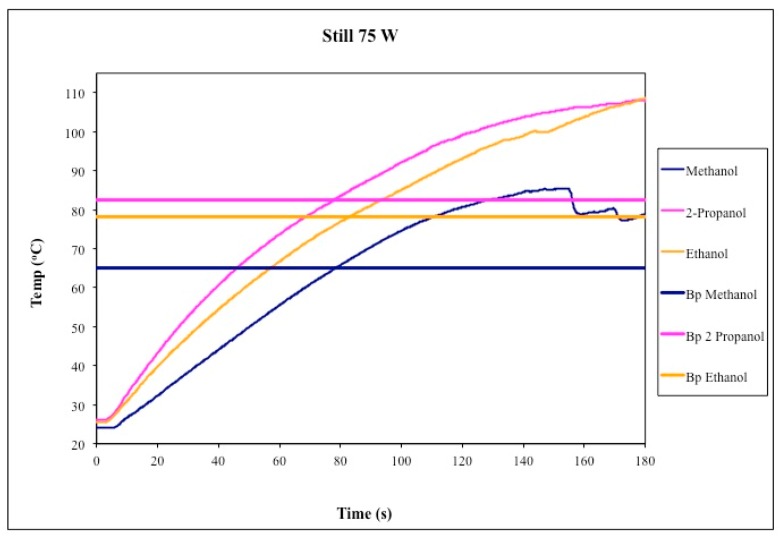
Temperature profiles of unstirred methanol, ethanol, and isopropyl alcohol under microwave heating for 3 min. The accepted boiling point of each liquid is plotted for comparison. The measured temperature of the liquid exceeded its normal boiling point in each case.

**Figure 3:**
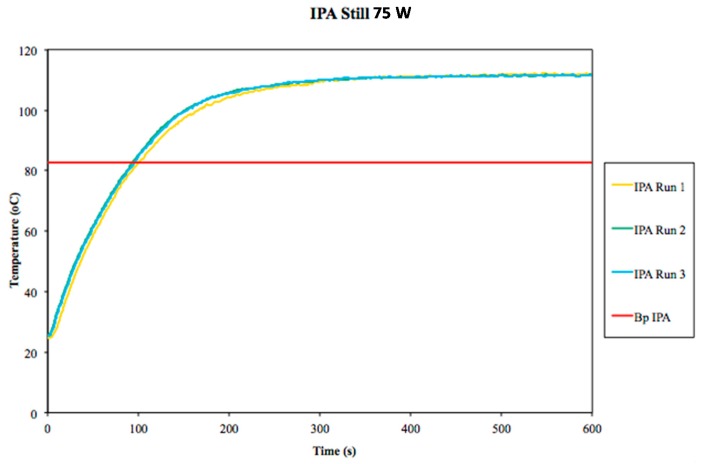
Temperature profiles of unstirred isopropyl alcohol (IPA) under microwave heating for 10 min (3 trials). The accepted boiling point of IPA is plotted for comparison.

The first set of heating experiments featured unstirred liquids. As shown in Figure 2, methanol, ethanol, and isopropyl alcohol were each heated using 75 W of applied microwave power for 3 min. In all cases, temperatures significantly above the normal atmospheric boiling solvent were recorded using the *in situ* fiber optic probe. The measured temperatures were sustainable and reproducible within a given set of experiments conditions (Figure 3). These data are consistent with the much more detailed studies of Mingos [17], Berlan [19], and Chemat [18], which we did not endeavor to duplicate in their entirety.

The exact magnitude of the deviation (ΔT) between the measured temperature of the liquid and its normal boiling point depends on several factors including volume of the liquid, size and shape of the flask, applied microwave power, and additives such as boiling chips, stir bar, or even a fiber optic probe. Our specific experimental design produced measurable bulk liquid temperatures for methanol, ethanol, and isopropyl alcohol that exceeded their normal boiling points by ΔT of 14 °C, 21 °C, and 28 °C above the expected values, respectively. Mingos previously measured analogous deviations of ΔT = 19 °C, 24 °C, and 18 °C for the same three solvents, but using larger volumes and a multimode (domestic kitchen) microwave oven to heat the liquids [17]. Berlan observed a lesser degree of superboiling, on the order of ΔT = 6 °C for methanol and 12 °C for ethanol, using smaller volumes (*ca.* 10 mL) of solvent and lower applied microwave power (*ca.* 40 W) [12]. Finally, Chemat reported superboiling of methanol and ethanol at ΔT = 14 °C and 11 °C [11], respectively, although these data were measured by as external IR sensor as opposed to an internal fiber optic probe.

Our interpretation of these collective data is that superboiling is a real but erratic phenomenon. It reliably occurs under microwave heating of unstirred liquids, but the exact magnitude is difficult to predict and/or reproduce, which makes it problematic.

Chemat described several methods for reducing the magnitude of “super-heated boiling” (superboiling), including the addition of boiling stones, stirring, air bubble injection, sonication, and even the use of an internal fiber optic probe. Each of these perturbations to the system reportedly produces additional nucleation sites at which the liquid→gas phase-change can occur, resulting in an overall regression of the bulk liquid temperature to the normal boiling point. Of these, Chemat noted that the “action of stirring or boiling stones is more severe and practically removes all super-heating.” In contrast, however, Kappe recently reported superboiling a toluene solution to ΔT of *ca.* 10 °C above its expected boiling point “even applying vigorous stirring” [14]. Our observations are largely in line with those of Chemat, although the size of the stir bar plays a role, as follows.

Our second series of experiments addresses the question of whether or not superboiling can reliably be observed in stirred liquids. We previously determined that superboiling was not a factor in our published experiments aimed at comparing reaction rates under conventional and microwave heating (*cf.* Figure 1, above) [9]. In those studies, our objective was to maintain consistent bulk temperature between conventional- and microwave-heated experiments. To that end, we used a stir bar commensurate with the size of the reaction vessel, and we visually monitored the reflux behavior to ensure that boiling was qualitatively similar between the two heating methods. Here, our objective is the opposite: to produce measurable superboiling in liquids stirred at the maximum rate allowed by the CEM microwave reactor. To this end, we chose a relatively small stir bar (10 mm × 3 mm) compared to the volume (30 mL) of the liquids we were examining.

**Figure 4:**
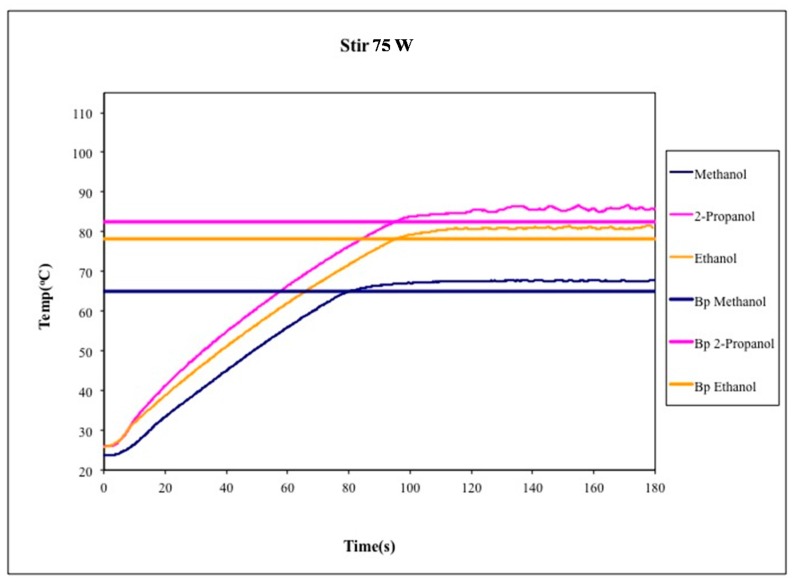
Temperature profiles of stirred methanol, ethanol, and isopropyl alcohol under microwave heating for 3 min. Liquids were stirred at the highest setting using a relatively small stir bar (10 mm × 3 mm). The accepted boiling point of each liquid is plotted as a straight horizon line for reference. The measured temperature (*in situ* fiber optic probe) of the liquid exceeded its normal boiling point in each case, although by less than what was observed in unstirred liquids.

**Figure 5:**
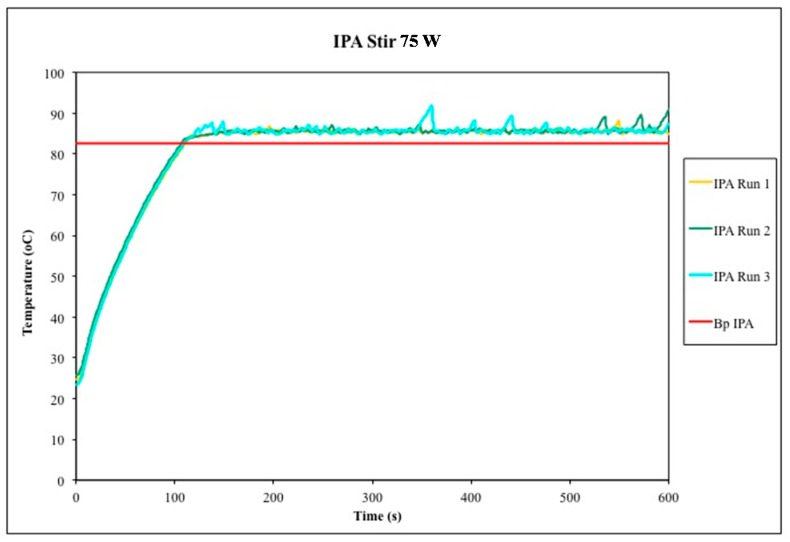
Temperature profiles for three experiments in which isopropyl alcohol (IPA) was subjected to microwave heating for 10 min, stirred at the highest setting using a relatively small stir bar (10 mm × 3 mm). The accepted boiling point of isopropyl alcohol is plotted for comparison. The measured temperature exceeded its normal boiling point by about 5 °C on average, with occasional and unpredictable spikes in temperature to *ca.* 10 °C above the boiling point.

As shown in Figure 4, superboiling can be observed in stirred methanol, ethanol, and isopropyl alcohol when using a relatively small stir bar, although the magnitude of ΔT was reduced to within *ca.* 5 °C of the normal boiling points. We then took an extended look at isopropyl alcohol (Figure 5), which is the liquid in which we observed the greatest magnitude of ΔT in the absence of stirring (up to 28 °C, Figure 2 and Figure 3, above). We saw occasional spikes in the measured temperature to as much as ΔT of *ca.* 10 °C above the boiling point, but these maxima were not sustained, predictable, or reproducible. On average, the measured ΔT for stirred isopropyl alcohol remained around 5 °C when using a relatively small stir bar in our experiments.

**Figure 6:**
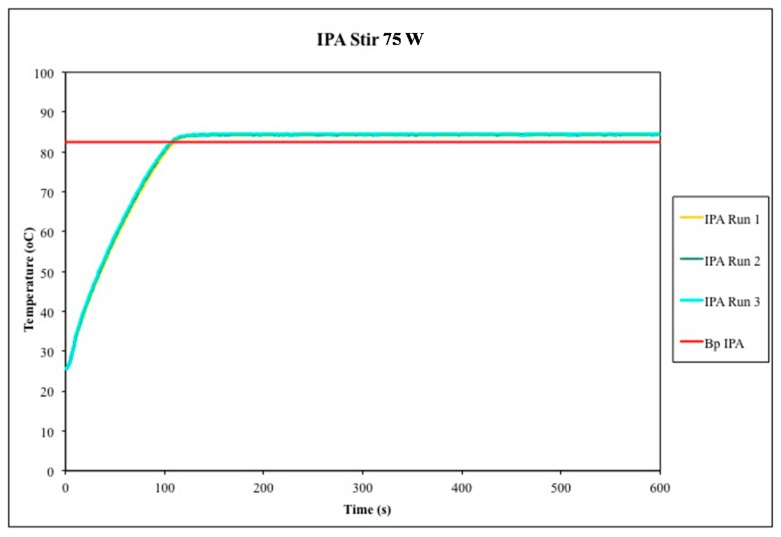
Temperature profiles for three experiments in which isopropyl alcohol was subjected to microwave heating for 10 min, stirred at the highest setting using a relatively large stir bar (25 mm × 5 mm). The accepted boiling point of isopropyl alcohol is plotted for comparison. The measured temperature exceeded its normal boiling point by about 2 °C on average.

When we switched to a larger stir bar (25 mm × 5 mm, Figure 6), the magnitude of ΔT dropped further to *ca.* 2 °C of the normal boiling point, and the occasional spikes in temperature were no longer observed, as reported previously by Chemat. Finally, we note that boiling chips—in our case, made of ground quartz glass—also reduced the magnitude of ΔT to with 1–2 °C of the normal boiling point (*cf.* Figure 1, above), whether used alone or in concert with stirring.

**Figure 7:**
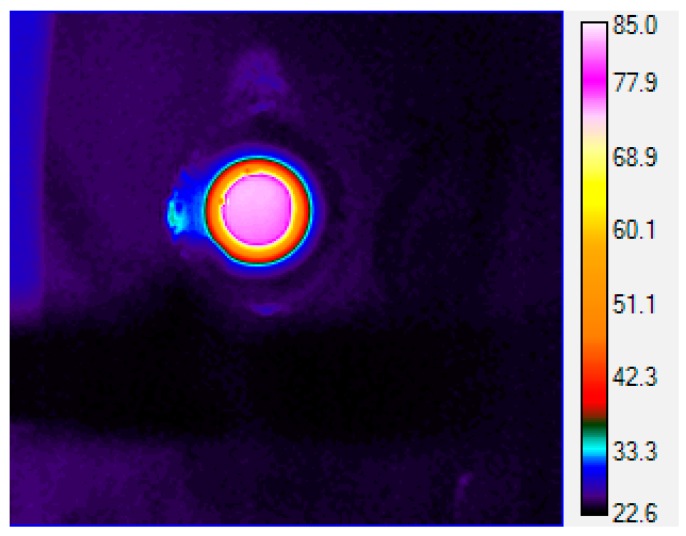
Thermal image of the bulk liquid IPA while at reflux with stirring under microwave heating at 75 W of power.

All of our temperature determinations for this superboiling study were made using a fiber optic thermometer immersed in the liquid, so it was important to determine whether the presence of the fiber optic was causing additional nucleation and yielding lower superboiling temperatures. To do this we measured the internal temperature of the solution remotely using a thermal imaging camera. Thermal imaging cameras measure black-body radiation in the far infrared, typically between 8 and 12 μm. However, quartz or glass reaction vessels have optical cutoffs >4 μm. Therefore, we fabricated sample cells out of high-density polyethylene, which is transparent to infrared radiation in the range detected by the camera. The measured temperature of ≤85 °C from the thermal imaging camera (Figure 7) was found to be in good agreement with the measured bulk temperature (≤85 °C) from the fiber optic probe, which is within about 2 °C of the expected boiling point for isopropyl alcohol (83 °C). In short, the presence of the fiber optic probe did not appear to affect the boiling temperature.

Our interpretation is that one can achieve superboiling in stirred liquids, although not nearly to the same magnitude as in unstirred liquids, provided that a relatively small stir bar is used, and other potential nucleation sites are minimized. More importantly (e.g., for comparing rates of reactions in refluxing solvents heated conventionally *vs.* using microwave energy), one can mitigate superboiling by ensuring vigorous stirring with an appropriately sized stir bar. However, what constitutes “a relatively small stir bar” *vs.* “an appropriately sized stir bar” likely depends on the volume and identity of the liquid, size and shape of the flask, magnitude of applied microwave power, and other reaction variables that may not always be significant on a laboratory scale.

## 4. Conclusions

Much of the early microwave chemistry literature has been called into question—sometimes appropriately—due to concerns over irreproducibility and underestimation of bulk solution temperatures. Indeed, chemical reactions heated by microwave radiation are sensitive to variables that synthetic chemists are not necessarily accustomed to considering on a laboratory scale. In spite of this, dedicated microwave reactors have become standard equipment in many modern synthesis labs. Routine users benefit from careful and thoughtful assessments of thermal events that are or are not likely to be possible using microwave dielectric heating, but it may be difficult for the casual reader to separate “myth from reality” [20] when reading microwave chemistry literature.

Based on our experiments, we conclude that the published literature accounts of microwave-assisted superheating and of the microwave-specific effect of nucleation-limited boiling (“superboiling”) are qualitatively correct: (1) temporary *superheating* of unstirred liquids is facilitated by microwave heating, although it can also be achieved with conventional heating; (2) temporary superheating of liquids persists under carefully controlled conditions only until nucleation is initiated, at which point boiling commences with rapid and sometimes violent release of excess thermal energy; (3) sustained *superboiling* of unstirred liquids can be observed thermometrically (and visually: superboiling more chaotic than conventional reflux) under the action of microwave heating, particularly in the absence of appropriate nucleation sites; (4) rapid stirring and/or boiling chips effectively mitigate microwave-specific superboiling; however; (5) one cannot presume to know the precise refluxing temperature of a bulk liquid without considering the dynamic interplay of factors including pressure, volume, solute identity and concentration, and the abundance and distribution of nucleation sites. Solvent reflux is commonly used in organic chemistry as a means of controlling bulk solution temperature under a given set of experiments conditions. It can also be used as a convenient means of identifying microwave-specific thermal effects, provided that one does not over-interpret the results in the absence of complementary thermometric data and control experiments.
